# Evaluation of 340B prescription assistance program on healthcare use in chronic obstructive pulmonary disease

**DOI:** 10.1016/j.rcsop.2023.100295

**Published:** 2023-06-14

**Authors:** Leah M. Taliaferro, Sarah Dodson, Melissa C. Norton, Samuel Ofei-Dodoo

**Affiliations:** a Ascension Via Christi Hospitals Wichita, Inc., 929 N Saint Francis, Wichita, KS 67214, United States; b Ascension Via Christi Hospitals, 1 Mt Carmel Pl, Pittsburg, KS 66762, United States; c University of Kansas School of Medicine - Wichita, 1010 N Kansas, Wichita, KS 67214, United States

**Keywords:** Drug costs, Pharmacy, Pulmonary disease, chronic obstructive, United States Health Resources and Services Administration, Vulnerable populations, 340B prescription assistance program

## Abstract

**Background:**

The federal 340B drug program was designed to stretch scarce federal resources to provide more comprehensive services for more eligible patients. To help satisfy community needs, 340B Prescription Assistance Programs (PAPs) allow eligible patients to access medications at significantly reduced costs.

**Objectives:**

To measure the impact of reduced-cost medications for chronic obstructive pulmonary disease (COPD) through a 340B PAP on all-cause hospitalizations and emergency department visits.

**Methods:**

This multi-site, retrospective, single-sample, pre-post cohort study involved patients with COPD who used a 340B PAP to fill prescriptions for an inhaler or nebulizer between April 1, 2018, and June 30, 2019. Data from included subjects were evaluated and compared in the year before and after each individual patient's respective prescription fill in the 340B PAP. The primary outcome evaluated the impact of 340B PAP on all-cause hospitalizations and emergency department visits. Secondary outcomes evaluated the financial impact associated with program use. Wilcoxon signed-rank test was utilized to assess changes in the outcome measures.

**Results:**

Data for 115 patients were included in the study. Use of the 340B PAP resulted in a significant reduction in the composite mean number of all-cause hospitalizations and emergency department visits (2.42 vs 1.66, Z = −3.12, *p* = 0.002). There was an estimated $1012.82 mean cost avoidance per patient due to reduction in healthcare utilization. Annual program-wide prescription cost savings for patients totaled $178,050.21.

**Conclusions:**

This study suggested that access to reduced-cost medications through the federal 340B Drug Pricing Program was associated with a significant reduction in hospitalizations and emergency department visits for patients with COPD, decreasing patients' utilization of healthcare resources.

## Highlights

• Prescription drug cost is a barrier to obtaining medications and maintaining adherence.
• The 340B Prescription Assistance Program offers discounted prescriptions, improving access.
• Improved access to medications results in cost avoidance to the healthcare system.
• The 340B Drug Pricing Program is an important tool to improve health and outcomes.

## 1 Introduction

One of the most significant problems facing healthcare today is poor medication adherence.^1^ Although many factors contribute to poor adherence to medications, a significant portion of adherence issues can be attributed to the high cost of medications.^2^, ^3^, ^4^ Karter and colleagues (2018) in a study of diabetes medications documented that patients who have to pay $20.00 or more are over two times less likely to pick up a medication from the pharmacy compared to patients who receive their medications at no cost.^5^ This problem is magnified for the 27.5 million Americans who are uninsured, as a single prescription can cost hundreds of dollars each month, limiting reliable and consistent access to medications.^6^

Chronic obstructive pulmonary disease (COPD) is defined as “persistent respiratory symptoms and airflow limitation that is due to airway and/or alveolar abnormalities usually caused by significant exposure to noxious particles or gasses” and presents as dyspnea, cough and/or sputum production.^7^ The Centers for Disease Control and Prevention (CDC) reports that 16 million Americans suffer from COPD.^8^ Medication options for COPD treatment are largely limited to inhaled medications delivered via inhaler or nebulizer devices, which are often high cost medications that lead to significant out-of-pocket expenses for patients. For example, in July 2020, the cash price of a common COPD medication used in certain patients with moderate to very severe disease, a fluticasone/salmeterol inhaler, was approximately $350 for a one-month supply. Patients with prescription insurance may still have significant out-of-pocket copays for many prescribed COPD medications. In addition to COPD being a cost-burdensome disease, the Centers for Medicare & Medicaid Services (CMS) Hospital Readmissions Reduction Program (HRRP) tracks readmission rates for several disease states, including COPD. Reimbursement for Medicare patients who are readmitted for COPD and other targeted disease states is significantly reduced as an incentive to hospitals to improve delivery of care for these diseases which historically have high readmission rates. The penalties that come with COPD hospital readmissions makes COPD care a focused metric to improve quality of care for many hospital systems.^9^

In 1992, congress enacted section 340B of the Public Health Service Act (PHSA), which is commonly referred to as the 340B Drug Pricing Program and operates under the Health Resources and Services Administration Office of Pharmacy Affairs (HRSA OPA). The intent of the federal 340B Drug Pricing Program is to allow eligible entities to stretch scarce federal resources as far as possible, reaching more eligible patients and providing more comprehensive services.^10^ Eligible entities participating in the 340B Drug Pricing Program are referred to as covered entities. The 340B statute requires pharmaceutical manufacturers who have a pharmaceutical pricing agreement (PPA) with Health and Human Services (HHS) and participate in Medicaid to offer a 340B ceiling price on covered outpatient drugs to covered entities, allowing eligible organizations to purchase discounted outpatient drugs for eligible patients.^11^ Patients are considered 340B eligible if they are a patient of the covered entity. Per program requirements, 340B discounted drugs may only be used for eligible patients. Section 340B(a)(4) of the Public Health Service Act specifies which covered entities are eligible to participate. These include HRSA-supported health centers and look-alikes, Ryan White clinics and State AIDS Drug Assistance programs, Medicare/Medicaid Disproportionate Share Hospitals, children's hospitals, and other safety net providers. Other eligible organizations include Federal grantees from HRSA, the Centers for Disease Control and Prevention (CDC), the Department of Health and Human Services' Office of Population Affairs, and Indian Health Services. All eligible entities must register with HRSA OPA to participate, and once registered, must comply with all 340B program requirements.^11^^,^^12^ Each covered entity has the flexibility to determine how savings realized through the 340B program will be used based on need in the communities they serve. Funding is often used for a variety of services, such as increased behavioral health services, free care for uninsured patients, or medication therapy management programs.^13^^,^^14^ In addition, 340B savings support the healthcare system and the patients they serve without being funded by taxpayer money. The 340B savings realized by the organization in this study are used to provide more comprehensive services to patients of the covered entity by both funding services tailored to the needs of patients, and also by passing savings on to eligible patients in the form of free or reduced-cost medication, referred to as the 340B Prescription Assistance Program (PAP). Programs such as the 340B PAP are made possible within the organization due to participation in the federal 340B Drug Pricing Program.

Currently, 340B research focuses primarily on highlighting the clinical programs that are funded by a participating covered entity's 340B savings. A recent comparison of 340B and non-340B hospitals found on average a significantly higher level of medication access services were provided at 340B hospitals compared to non-340B hospitals as a result of the services funded through 340B program savings.^15^ For example, Jones and colleagues (2019) highlighted a primary care-based hepatitis C virus treatment program that was only feasible due to partial 340B funding.^16^ Similarly, Wu and colleagues (2019) described how 340B funding allowed an institution to provide a financially sustainable bedside medication delivery service to ensure hospitalized patients received medications prior to discharge.^17^ A study by Castellon and colleagues (2014), however, evaluated a different approach to 340B program savings by showing the financial impact of a 340B PAP on improving medication cost by demonstrating significant medication savings provided to uninsured patients.^18^ While these studies are vital in demonstrating the importance of the 340B program, current literature does not directly evaluate the effects of 340B PAPs on healthcare utilization. Furthermore, the most recent literature describing 340B PAP cost savings highlights data from 2014.^18^ This study evaluated the impact of a 340B PAP both on healthcare utilization and cost savings in patients with COPD. Despite the 340B program showing benefit to patients, there are actions by drug manufacturers and discussions among lawmakers of significantly reducing the program's reach, leaving vulnerable citizens with less access to the medications and quality healthcare.^14^ This study helps to add to the growing literature needed to justify the importance of 340B programs for vulnerable populations.

## 2 Methods

This multi-site study focuses on a service, referred to as the 340B PAP. This service is provided by a healthcare system in a midwestern state in the United States (US) with study sites located in a large urban city and a small rural town. Within the large urban city site, study locations included two community teaching hospitals, two family medicine clinics, a transitions of care clinic and a heart failure clinic. Study locations in the smaller rural town included one hospital and pulmonology clinic. At the time of the study, the health system had two 340B entities with the two urban hospitals qualifying as Disproportionate Share Hospitals and a rural hospital qualifying as a Rural Referral Center. Each entity had affiliated outpatient clinics which served as 340B child sites that provided a variety of services and covered a range of specialties. The health system offered the 340B PAP through both hospital-owned outpatient pharmacies as well as an external 340B contract pharmacy. Both study locations had considerable underserved populations. According to information collected from the United States Census Bureau in July 2020, the large urban city is the largest city in this midwestern state, and 16.2% of citizens live in poverty, while 14.4% of citizens below the age of 65 years old live without health insurance. The small rural town is located where 29.5% of citizens live in poverty and 15.1% of citizens below the age of 65 years old live without health insurance.^19^ This study was approved by the affiliated institutional review boards as exempt from full review.

In 2018, the health system in this study began piloting a 340B PAP in a large urban midwestern city and smaller rural midwestern town. The 340B PAP within the health system allowed patients to access medications at significantly reduced prices at the hospital-owned or contract pharmacy. The program focused on disease states in which medications are historically cost restrictive for patients and access to these medications may have a significant impact on patient outcomes. Utilizing the reduced priced medications available through the federal 340B Drug Pricing Program, the 340B PAP allowed the health system to pass medication cost savings directly to patients. For example, the out-of-pocket patient cost for the aforementioned fluticasone/salmeterol inhaler was approximately $350 per month without insurance coverage, however through the 340B PAP, the out-of-pocket cost for an eligible patient was approximately $15 for a three-month supply. A patient was considered eligible for the health system's 340B PAP if they were a patient of the 340B covered entity and met the definition of a patient as defined by the Health Resources and Services Administration.^20^

A multi-site, retrospective observational cohort study designed to compare the mean rate of all-cause hospitalizations and emergency department visits per patient before and after 340B PAP use with one single group of patients serving as their own control was used. Patients were retrospectively screened for inclusion into the study and included over a 15-month period with additional demographic and clinical data collected one year prior to and one year following the patient's respective inclusion in the study.

The primary outcome was the mean number of all-cause hospitalizations and emergency department visits before and after 340B PAP use. Secondary outcomes included a comparison of mean rate of 30-day hospital readmissions, estimated cost avoidance due to reduction in the frequency of healthcare use, program-wide medication cost savings after utilization of 340B PAP, and a comparison of patient medication costs. Costs of medications for comparison purposes were established using the wholesale acquisition cost (WAC) for each medication at the end of the study period. Medication savings were calculated utilizing the difference between WAC price and 340B PAP out-of-pocket patient cost. Additionally, estimated cost avoidance due to reduction in the frequency of healthcare use was calculated by multiplying each respective mean reduction in emergency department visits and hospitalizations by the respective mean direct costs of COPD-related emergency department visits or hospitalizations within the health system.

Data were retrospectively collected within the previously listed study locations from two sources: 1) electronic health records (EHR), and 2) prescription fill records. Patients who first filled a prescription for an inhaler or nebulized medication at the hospital-owned or contract pharmacy through the 340B PAP between April 1, 2018, and June 30, 2019, and had a documented ICD−10 (International Classification of Disease, Tenth Edition) code for COPD at either a clinic visit, emergency department visit or hospitalization were included in the study. Any patients who were prisoners, pregnant or <18 years of age were excluded. Patient data from the one-year period before and after each patient's respective first 340B PAP fill were collected and evaluated with a total data collection time frame of April 1, 2017, and June 30, 2020.

**Fig. 1:**
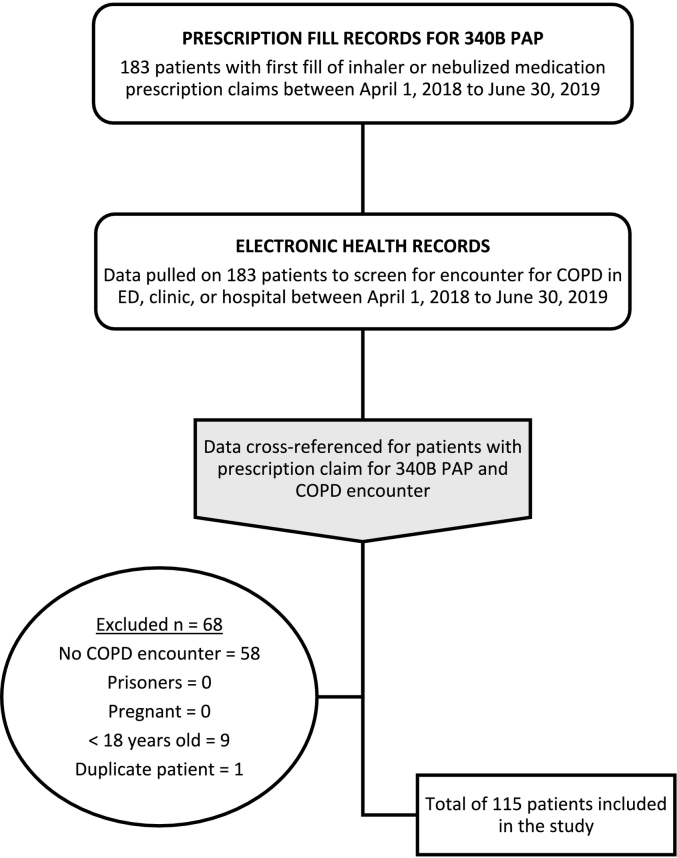
Patient selection process.

Patients were initially identified using a report of prescription claims filled for the first time through the 340B PAP for inhalers or nebulized medications during the study period. Data were then extracted for the initially identified patients through each covered entity's EHR and screened for COPD ICD-10 code, J44 and all affiliated subcodes, and inclusion and exclusion criteria (See Fig. 1). Once study patients were identified, demographic and clinical data were extracted from the EHR and prescription fill records were extracted from a 340B report of prescription claims from the one-year period before and after each patient's respective first 340B PAP prescription dispense date. COPD-related medications included any inhaled or nebulized medications. Baseline characteristics and demographic data were obtained from the EHR documentation dated closest to each patient's first 340B PAP fill. Hospitalizations and emergency department visits were evaluated in the year before and after each patient's first respective 340B PAP fill. Additional clinical data were collected on COPD-related comorbidities using appropriate ICD-10 codes and included chronic kidney disease, chronic lower respiratory diseases (in addition to COPD), hypertension, ischemic heart disease, nicotine dependence, and non-ischemic heart diseases.

Data were expressed as mean ± standard deviation (SD) and screened for outliers, and assumptions of normality and homoscedasticity. Outliers and normality assumptions were tested using histograms, skewness, and kurtosis. Assumptions of homogeneity of variance and sphericity were evaluated using Levene's and Mauchly's tests, respectively. Frequency distributions were calculated to examine the financial savings as a result of 340B PAP use, and to create a demographic profile of the participants. Wilcoxon signed-rank test was used to determine the mean rate of emergency department visits, all-cause hospitalizations, and 30-day hospital readmission before and after 340B PAP use. A sample size of 34 was calculated as necessary for adequate power (>0.80) to detect significant group differences among the variables with 0.05 effect size. All analyses were 2-sided with alpha of 0.05. The IBM SPSS (Statistical Package for the Social Sciences), version 26 was used for these analyses.

## 3 Results

A total of 183 patients were retrospectively screened for inclusion in the study. After application of inclusion and exclusion criteria, data on 115 patients were included in the study. As Table 1 shows, the average age of patients was 60.7 years (SD = 10.5); 53.9% were female; and the majority (80.6%) were White or Caucasian. Forty percent of patients had Medicare insurance coverage; 47% were current smokers; over 52% had hypertension; 45% had a chronic lower respiratory disease in addition to COPD. Regarding inhaled medications, 57% of patients used the program to fill a prescription for a short-acting beta agonist, and 52% used the program to fill a prescription for an inhaled corticosteroid and long-acting beta agonist combination product.

**Table 1:** Participants' characteristics at baseline (*N* = 115).

Female, No. (%)	62 (53.9)
Age, y, mean (SD)	60.7 (10.5)
Race, No. (%)	
White or Caucasian	95 (82.6)
Black or African American	16 (13.9)
American Indian or Alaska Native	1 (0.9)
Asian	1 (0.9)
Unknown	2 (1.7)
Smoking Status, No. (%)	
Never	10 (8.7)
Current	54 (47.0)
Former	51 (44.3)
Body Mass Index, kg/m^2^, mean (SD)	29.1 (8.8)
Insurance Status, No. (%)	
Medicare	46 (40.0)
Medicaid	20 (17.4)
Commercial	10 (8.7)
Uninsured	39 (33.9)
Comorbidities, No. (%)	
Chronic kidney disease	6 (5.2)
Chronic lower respiratory disease (in addition to COPD)	52 (45.2)
Hypertension	60 (52.2)
Ischemic heart disease	27 (23.5)
Nicotine dependency	26 (22.6)
Non-ischemic heart disease	29 (25.2)
Inhaled Medications, No. (%)	
Short-acting beta agonist	65 (56.5)
Inhaled corticosteroid	6 (5.2)
Long-acting beta agonist	3 (2.6)
Inhaled corticosteroid/long-acting beta agonist	60 (52.2)
Long-acting muscarinic antagonist	35 (30.4)
Long-acting muscarinic antagonist/long-acting beta agonist	1 (0.9)

The Wilcoxon signed-rank tests showed 340B PAP use elicited a significant reduction in the primary outcome, composite number of all-cause hospitalizations and emergency department visits, from 2.42 ± 2.93 during the pre-340B PAP year to 1.66 ± 2.18 during the post-340B PAP year (Z = −3.12, *P* = 0.002; Table 2). Only emergency department visits were significantly reduced, from 1.37 ± 2.20 pre-340B PAP to 0.70 ± 1.24 post-340B PAP (Z = −3.42, *P* = 0.001; Table 2).

**Table 2:** Clinical outcomes (*N* = 115).

	Pre-340B		Post-340B			
Outcome	Mean	SD	Mean	SD	*Z*	*P* value
Total ED visits and all-cause hospitalizations	2.42	2.93	1.66	2.18	−3.12^a^	0.002
Emergency department visits	1.37	2.2	0.74	1.24	−3.42^a^	0.001
All-cause hospitalizations	1.04	1.31	0.92	1.52	−1.06^a^	0.291
30-day hospital readmissions	0.23	0.69	0.31	0.94	−0.76^b^	0.45

Abbreviation: ED, emergency department.

a Based on negative ranks.

b Based on positive ranks.

Financial outcomes are represented in Table 3. The estimated mean cost avoidance per patient due to reduction in healthcare utilization was $1012.82. The total prescription out-of-pocket expense reduction for all 115 patients through the 340B PAP was $178,050.21 annually, representing a savings of $14,837.52 monthly. The mean amount of prescription out-of-pocket expense saved annually per patient through the 340B PAP was $1548.26. The mean amount of out-of-pocket expense saved per prescription per patient through the 340B PAP was $622.55.

**Table 3:** Financial outcomes.

Outcome	Total
Annual program-wide cost savings	$178,050.21
Monthly program-wide cost savings	$14,837.52
Mean annual savings per patient	$1548.26
Mean savings per prescription	$622.55

## 4 Discussion

This study demonstrated a statistically and clinically significant reduction in the composite mean number of emergency department visits and hospitalizations. It was noted that over 50% of patients evaluated filled a prescription for an inhaled corticosteroid and long-acting beta agonist combination product. However, appropriateness of COPD medications was not evaluated, as it was not within the scope of this study. The results of this study revealed that patients utilizing cost savings for COPD medications through the 340B PAP showed a significant reduction in mean composite number of emergency department visits and hospitalizations compared to pre-340B PAP utilization. Although there was a statistically significant reduction in ED visits, the lack of a statistically significant reduction in hospitalizations and the increase in 30-day hospital readmissions may be related to a variety of causes including disease progression, hospitalizations for other causes, or medication nonadherence.

While many of the financial benefits of 340B PAPs are well known and easily calculable, our study went beyond common financial markers and evaluated cost avoidance based on decreased healthcare utilization. Due to the decrease in healthcare utilization, there was an estimated mean reduction in direct care cost of $1012.82 per patient within a one-year period in addition to the savings from reduced medication acquisition costs.

To our knowledge, no previous studies have evaluated a 340B PAP or other prescription assistance program's effects on healthcare utilization and provides further justification of the benefit the 340B program can have on a disease considered high risk for hospital readmissions and one which is cost-burdensome to treat.^9^ Historically, evaluations such as the Asheville project demonstrated how initial investments in care, community-based pharmaceutical care services for patients with diabetes in this instance, can have significant impacts on limiting utilization of more costly forms of care.^21^ From a healthcare utilization standpoint, we evaluated mean cost avoidance per patient due to reduction in healthcare utilization (emergency department visits or hospitalizations) and found an estimated mean reduction in direct care cost of $1012.82 per patient during the one-year post-340B PAP study period.

In addition to mean cost avoidance, our results provide additional, more recent data to highlight 340B PAP cost savings for patients, similar to Castellon's evaluation.^18^ However, Castellon and colleagues (2014) evaluated patients receiving any medications through a 340B program and used drugstore.com as a price comparator, whereas our study specifically evaluated inhaled medications for COPD and used WAC as a price comparator. Our results showed a mean savings per prescription of $622.55, whereas the Castellon evaluation showed only a mean saving of $62.31 per visit.^18^ The differences in prescription savings between the two studies is likely attributed to both the price comparator utilized as well as shifting drug prices between the time periods of each study.

Some limitations of our study include the coronavirus disease 2019 (COVID-19) pandemic, utilization of all-cause hospitalizations and emergency department visits, inability to assess adherence, its retrospective nature, as well as being a single health system study. The COVID-19 pandemic reached the central US, where this study took place, during March 2020. It is difficult to truly assess the impact the pandemic had on the last four months of the study period as hospitalizations decreased across the nation, however patients with COPD were at an increased risk for severe COVID-19 that could potentially result in hospitalization and poor outcomes due to their COPD.^22^ In utilizing an all-cause visit outcome, healthcare visits unrelated to COPD could have confounded study results. However, it is well known that hospitalizations and emergency department visits are often multifactorial, and for this reason, we felt it was important to include all-cause visits.^23^ Another limitation is the inability to determine days of therapy coverage and adherence to 340B PAP medications. In addition, patients may have been provided the 340B PAP medications as hospital discharge prescriptions, which is most often a 3-month supply with no additional refills. Total days covered or proportion of days covered by the medication fills and any subsequent refills, if available, was not evaluated. Lastly, as commonly occurs in retrospective studies, data evaluation was limited by a single system EHR and prescription fill history, as well as a lack of knowledge of patients' care outside the health system.

The illustrated clinical and financial benefits as well as some of the limitations of our analysis raise thoughts and questions that warrant further evaluation. One significant question is the role that medication adherence plays in 340B PAPs and overall clinical outcomes; further studies are needed to gain additional insight. This study included utilization of a 340B contract pharmacy in a rural midwest community as a mechanism to offer patients access to the 340B PAP, providing a significant benefit and medication access to this underserved community. Unfortunately, 340B contract pharmacy arrangements are often heavily scrutinized and restricted, most commonly by pharmaceutical drug manufacturers. In July 2020, six major drug manufacturers unlawfully stopped providing discounted pricing to 340B contract pharmacies over concerns that the patients are not receiving the benefit of the 340B program.^24^ Since then, numerous other drug manufacturers have followed suit, which has resulted in several court cases with mixed rulings to date. In April 2022, the American Hospital Association (AHA) conducted a survey to determine the impact of limiting 340B pricing to contract pharmacies. More than 300 hospitals and health systems were included in the survey, demonstrating significant average annual financial losses to critical access hospitals (CAH) and disproportionate share hospitals (DSH) of $507,000 and $2,960,000, respectively. In addition, CAHs reported on average, 44% of their total 340B savings coming from contract pharmacies, with several CAHs reporting all of their 340B savings coming from these arrangements.^25^ However, the utilization of 340B contract pharmacies provide covered entities an additional avenue to reach patients and help to satisfy the intent of the federal 340B Drug Pricing Program. As it pertains to this study, the smaller, rural study location did not have a hospital-owned pharmacy and relied on a contract pharmacy as the mechanism to provide free and discounted medications to eligible patients. Given the drug manufacturer restrictions that have been imposed since July 2020, replicating the outcomes of our study may prove challenging for covered entities that do not have a pharmacy owned by the covered entity that is capable of dispensing prescriptions.

Our findings demonstrate the advantages of utilizing the 340B Drug Pricing Program for vulnerable populations and provides further justification for continuation and growth of 340B PAPs. Results demonstrated this program reduced the cost of medications, and suggests that 340B PAP use can also improve clinical outcomes by reducing the number of all-cause emergency department visits and hospitalizations. Based on these results, clinicians should be encouraged to increase utilization of 340B PAPs, when possible, to improve the care of their patients. Additionally, health systems should utilize 340B funds to expand existing medication assistance programs to provide more accessible and sustainable healthcare solutions for patients. The 340B program is a vital tool to provide care to those in need by mitigating the immediate and long-term impacts of high and rising drug prices and helps covered entities to provide necessary access to care and medications to those most vulnerable. While the drug discounts provided to covered entities represent only 3.6% of the total US drug market, they make a significant impact on helping to enhance services for those most in need.^14^ In addition, the 340B program enables safety net providers not only to mitigate high and rising drug prices, but to be good stewards of finite patient, program and provider resources with a goal to improve the health of the patients they serve. Furthermore, our study adds to the support of previous literature demonstrating the positive impact that programs partially funded through 340B program savings can have on underserved patient populations by illustrating the positive impact that a 340B PAP can have on the COPD population. It is vital to these patient populations that the federal 340B Drug Pricing Program continues and access to medications within 340B PAPs is not limited.

## Declaration of Competing Interest

The authors declare the following financial interests/personal relationships which may be considered as potential competing interests:

The authors disclose financial support received through 340B Health for research and/or publication of this work. The funders had no role in study design, data collection and analysis, or preparation of the manuscript.
